# Preferences of experts and the general public about wildlife management in Spain

**DOI:** 10.1007/s13280-025-02280-z

**Published:** 2025-10-23

**Authors:** Daniela Alba-Patiño, Miguel Delibes-Mateos, María Martínez-Jauregui, Rafael Villafuerte Jordán, Beatriz Arroyo-Lopez, Jenny Anne Glikman, Mario Soliño

**Affiliations:** 1 Department of Biology and Geology, Centro Andaluz para el Cambio Global –Hermelindo Castro (ENGLOBA), University of Almería, Carretera Sacramento, S/N, 04120 Almería, Spain; 2 Instituto de Estudios Sociales Avanzados (IESA-CSIC), Plaza Campo Santo de los Mártires, 7, 14004 Córdoba, Spain; 3 Centro de Investigación Forestal (INIA-CSIC), Carretera La Coruña, 7.5 Km, 28040 Madrid, Spain; 4 Instituto de Investigación en Recursos Cinegéticos (IREC, CSIC-UCLM-JCCM), Ronda de Toledo 12, 13005 Ciudad Real, Spain; 5 Instituto de Investigaciones Marinas, CSIC, Rúa Eduardo Cabello 6, 36208 Vigo, Spain

**Keywords:** Biodiversity conservation, Birds, Mammals, Online questionnaire, Overabundant species, Scarce species

## Abstract

Biodiversity loss is a global challenge that requires conservation policies integrating ecological, social, and economic considerations. While scientific evidence guides policy decisions, public preferences can also shape strategies, sometimes diverging from optimal ecological outcomes. This study examines the alignment between expert and public preferences regarding wildlife management in Spain, one of Europe’s most biodiverse countries. Using a discrete choice experiment conducted among experts attending the 2021 Spanish Society for the Conservation and Study of Mammals (SECEM) Conference, we compared their views with those of the Spanish public. Both groups prioritized managing scarce species over overabundant ones, though experts showed greater concern about human impacts on wildlife. Experts also exhibited more nuanced views on conservation beyond protected areas. Latent class modeling identified five behavioral classes within each group, highlighting substantial preference heterogeneity. Experts and the public also diverged in their willingness to pay for conservation efforts. These results emphasize the need to integrate expert knowledge with public values for socially acceptable and ecologically effective policies.

**Supplementary Information:**

The online version contains supplementary material available at 10.1007/s13280-025-02280-z.

## Introduction

High rates of habitat degradation and species loss show that global biodiversity is currently in crisis (IPBES 2019). One of the biggest challenges to address this nature crisis is designing and implementing effective environmental policies. As funds are limited, policy-makers have to prioritize which species or habitats are the main target of the policies. Such decisions are complex and often involve connections between social, scientific, and political concerns (Martín-López et al. 2009). Environmental policies are most effective when based on scientific evidence and public support (Eisner et al. 1995; Francis et al. 2005). However, policy decisions are often influenced by the preferences of the general public (Monroe 1979), which do not necessarily match the recommendations made by environmental experts. Examples of policy driven by societal factors include policies regulating the exploitation of wildlife species whose populations have sharply declined. A paradigmatic case is the European eel (*Anguilla anguilla*), whose conservation status is critical (Deinet et al. 2020), but its fishing is still allowed in different countries due to public demand. Furthermore, legal instruments are frequently biased toward charismatic, familiar, esthetic vertebrates, which may jeopardize certain biodiversity conservation programs (Carrete et al. 2022). Associated to an increase in mutualist values (characterized by viewing wildlife as part of the social community rather than in utilitarian terms), societal rejection of certain actions has at times influenced the tools implemented for wildlife management programs (Martínez-Jauregui et al. 2020; Ribeiro et al. 2021). In this context, assessing similarities and differences between the views and preferences of the general public and experts regarding wildlife and its management may provide useful insights for designing conservation policies that are evidenced-based and socially acceptable.

Discrete choice experiments (DCE) are widely used to estimate societal preferences for predefined management programs (Turner et al. 2007; Johnston et al. 2017). In a recent study using DCE, Martínez-Jauregui et al. (2023) showed that the preferences of the general public for basic wildlife management principles are very similar across 6 European countries, including Spain, one of the most biodiverse countries in Europe (Soler Luque and Kostecka 2018). In particular, Martínez-Jauregui et al. (2023) showed that managing scarce species is preferred by Europeans over managing abundant species, including those that are defined as ‘excessively abundant.’ This is unsurprising, given that over 1 million species are threatened with extinction (IPBES 2019), and the need for action to reverse such biodiversity loss is acknowledged by most of the European Society (European Commission 2018). However, some wildlife species like ungulates are rapidly increasing in Europe, mostly favored by land use changes and conservation policies, often reaching densities that negatively affect ecosystems and human livelihoods (Carpio et al. 2021). In such cases, intensive management is often needed to reduce the impacts of overabundant wildlife; for example, controlling wild boar (*Sus scrofa*) populations to prevent the spread of African Swine Fever (Palencia et al. 2023), reducing environmental impacts of overabundant ungulates in Spanish National Parks (Martínez-Jauregui et al. 2020; Martínez-Jauregui and Soliño 2021) or limiting the spread of invasive species (McDowell et al. 2014).

Similarly, research has shown the higher importance of certain habitats in the current era of biodiversity loss for maintaining overall ecosystem services (Mooney et al. 2009). Thus, several initiatives target the conservation of specific habitats, including, for example, the new EU forest strategy for 2030, the Ramsar convention of wetlands or the increased environmental emphasis of the Common Agricultural Policy for farmland habitats (Donald et al. 2002; Lier et al. 2022). Despite this, Martínez-Jauregui et al. (2023) found no clear public preferences for managing wildlife in specific habitats, excepting a more or less generalized rejection of intervening in peri-urban areas. In contrast, there was a clear preference to prioritize management in protected areas rather than in non-protected areas. Establishing protected areas is a key tool for the conservation of biodiversity (He and Wei 2023). However, these areas may also impact the well-being of local communities, often leading to conservation conflicts (Redpath et al. 2015), and there exists a debate within research literature about whether biodiversity conservation should rely on land sparing or land sharing (Fischer et al. 2014). Finally, Martínez-Jauregui et al. (2023) found a preference among the general public for programs that included payments for environmental services (PES) through programs that included economic compensation for landowners. This result suggests that PES may be perceived by the public as a fair and effective tool to support conservation, enhancing the legitimacy of wildlife management interventions and aligning individual and collective interests (Arriagada and Perrings 2013).

Here, our main objective was to explore whether those public’s preferences match those of wildlife experts, by using DCE to investigate experts' priorities in Spain. We expected that experts would be supportive of managing overabundant species and would prioritize managing wildlife in endangered habitats like those subject to the above-mentioned initiatives. To do so, we used a discrete choice experiment (DCE) to identify experts’ priorities, with particular attention to species status (scarce vs. overabundant), habitat type, protection status, and the role of payments for environmental services (PES) (Varela et al. 2014). We further explored heterogeneity within the expert group by applying latent class models, which allowed us to detect distinct preference patterns at both the attribute and population levels. In addition, we investigated experts’ perceptions of wildlife impacts—defined as situations where people negatively affect wildlife, or where wildlife negatively affects people’s well-being (Young et al. 2005, 2007)—as these perceptions may shape management preferences (Frank et al. 2015; Delibes-Mateos et al. 2020). Finally, we compared experts’ perceptions of wildlife impacts with those of the general public in Spain, as reported by Delibes-Mateos et al. (2023).

## Materials and methods

### Sampling strategy and questionnaire design

To assess experts’ preferences regarding wildlife management, we conducted an online questionnaire among the attendees of the XV Conference of the Spanish Society for the Conservation and Study of Mammals (SECEM), which was celebrated in December 2021. SECEM is an environmental non-governmental organization (NGO) that was founded in February 1991 and counts approximately 1000 affiliates. It is a member of the International Federation of Mammologists, the European Mammal Foundation, and the Spanish Committee of the International Union for Nature Conservation (IUCN). The main objective of SECEM is promoting knowledge and conservation of mammals through conducting studies and conservation activities. Since 1993, SECEM organizes a biannual conference focused on the study and conservation of mammals. An average of 300 people usually attend such conferences; 276 attended the 2021 conference in which this study is focused on. Attendees principally include researchers and wildlife technicians and managers with expertise in the study and conservation of wildlife, particularly mammals. During the 2021 conference, the study was advertised at the end of plenary talks and thematic sessions. A slide was projected on the screen explaining the aims of the study and encouraging attendees to respond. A QR code was available to access the survey using mobile phones. This information was also shown in a poster placed near the entrance to the conference rooms. A week after the end of the conference we sent an e-mail reminder to all attendees. The list of contacts was publicly available in the book of abstracts of the conference. Participation was voluntary and responses were anonymous. CSIC Ethic Board approved the protocol, certificate 020/2021.

**Fig. 1:**
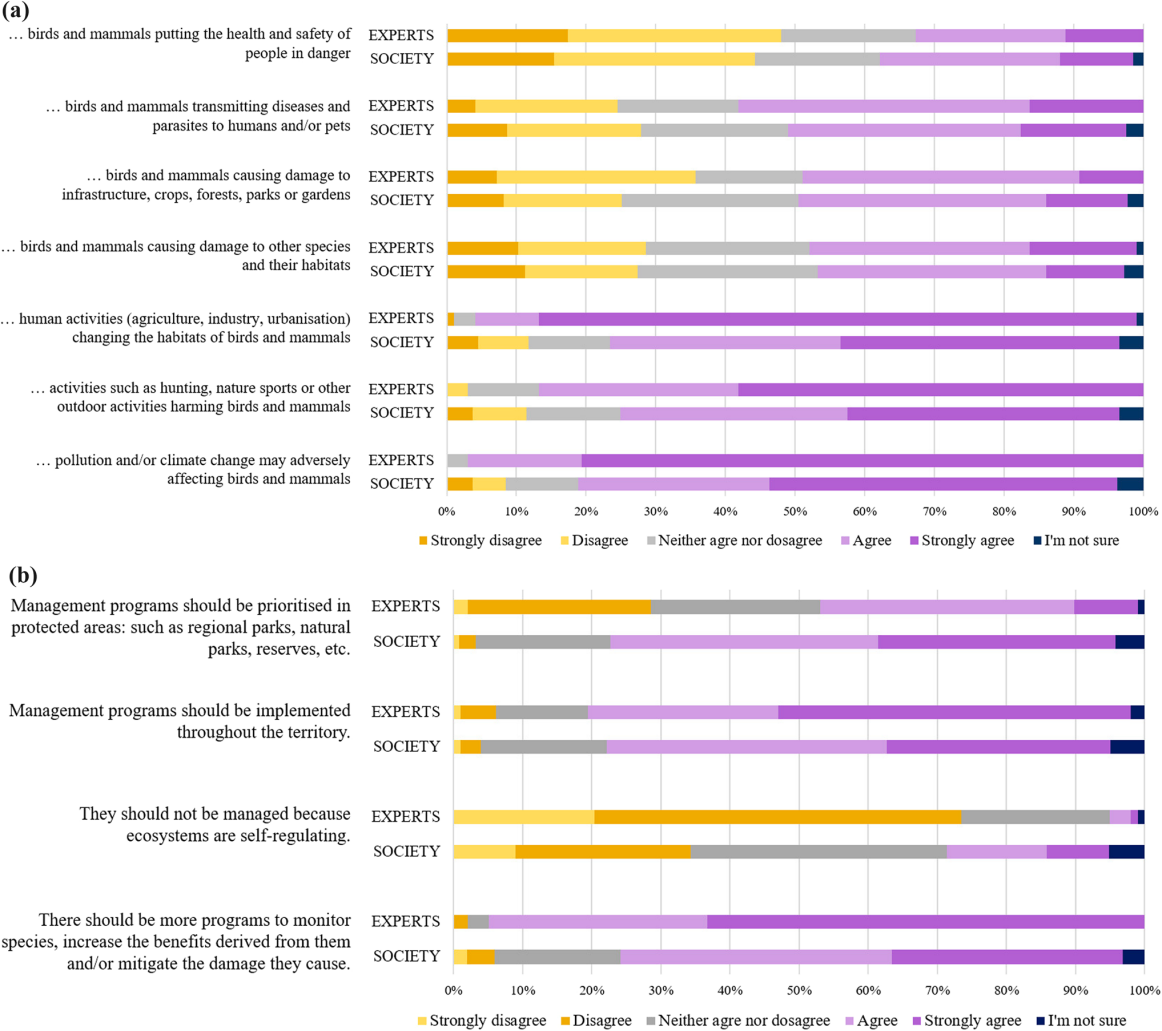
Percentage of agreement or disagreement by experts and Spanish society on statements about wild birds and mammals: a) impacts b) aspects of management programs

The questionnaire was posted in TickStat® website, and replicated a few parts of the survey conducted by Martínez-Jauregui et al. (2023) in six European countries to assess views and preferences of the general public regarding wildlife and its management. They focused on birds and mammals because these are usually better known by the general public, provide many ecosystem services to society, and receive more conservation funding (Martínez-López et al. 2009). The content of that questionnaire was pretested for understanding and coherence on several people in Spain with different backgrounds (wildlife technicians, wildlife researchers, and citizens) and a pilot of 40 Spanish citizens from a consumer panel was conducted to identify further potential drawbacks (Martínez-Jauregui et al. 2023). The questionnaire for experts included the DCE and a set of questions aimed at capturing how participants viewed wildlife impacts and management. The latter consisted on 11 statements with which participants had to rank their level of agreement in a 1–5 Likert scale (from strongly disagree to strongly agree). The option ‘I’m not sure’ was also available for when participants felt unable to express a clear opinion. Seven of these statements were related to wildlife impacts, including impacts of human activities, climate change and pollution on wildlife and impacts of wildlife on ecosystems, people and their livelihood (Fig. 1a, but see also Delibes-Mateos et al. 2023), and four addressed different aspects of wildlife management (Fig. 1b).

To design the DCE, we defined wildlife management programs based on a set of attributes and an associated cost to the respondent. The experimental design allowed all possible combinations of attribute levels, with no restrictions imposed, and was generated using a D-efficient design (Olsen and Meyerhoff 2016). Each respondent was presented with 12 choice cards, each showing three program alternatives and a ‘no intervention’ option. Respondents were reminded that opting out was a valid choice, and a follow-up question was included for those who consistently selected this option.

The attributes and levels used in the experiment are presented in Table 1. They were designed to reflect contrasting and policy-relevant aspects of wildlife management in the European context. One attribute described the population status of the species to be managed, distinguishing between rare or declining species and those that are overabundant or expanding—two widely recognized priorities in wildlife management across Europe (Caughley 1977; Krausman and Cain 2013). The habitat attribute included four types—forest, aquatic, agricultural, and peri-urban—selected for their ecological relevance and prominence in the landscape and in conservation strategies, such as the EU Habitats Directive, the Green Infrastructure Strategy, and the Spanish National Strategy for farmland birds (Directive et al. 1992; European Commission 2013; Ministerio para la Transición Ecológica y el Reto Demográfico 2022). The protection status attribute distinguished whether management would take place within or outside protected areas, reflecting debates on land sparing versus land sharing approaches (Fischer et al. 2014), and recent policy targets like protecting 30% of the territory by 2030 (Convention on Biological Diversity 2020). We also included the presence or absence of payments for environmental services (PES) to landowners, a policy tool increasingly recognized in agricultural landscapes, including in Spanish strategy for farmland birds (Ministerio para la Transición Ecológica y el Reto Demográfico 2022). Finally, cost was defined as an annual tax over the next five years to fund the program, allowing estimation of willingness to pay. Respondents were presented with an explanation of all the attributes and levels before the actual choice experiment. A brief summary of the rationale for the attribute selection is included in the Supplementary Information S1.

**Table 1:** Attributes and levels of attributes considered in the discrete choice experiment. Adapted from Martínez-Jauregui et al. (2023)

Attributes	Levels of the attribute
Situation of wild animals	Animals that grow in number and/or expand in terms of territory; animals that are scarce in terms of numbers and/or territory
Type of habitat	Peri-urban; Agricultural; Forest; Aquatic
Protection of the territory	Protected; Non-protected
Payment for environmental services	Payment; No payment
Cost	0€, 10€, 20€, 30€, 40€, 50€, 60€

An example of a management scenario presented to respondents might be: ‘A program aimed at controlling overabundant species in peri-urban areas outside protected zones, which includes PES to landowners and would cost €40 per year over the next five years.’ This type of combination allows us to examine experts’ preferences across a range of plausible, policy-relevant situations. A more detailed description of the DCE design and examples of specific choice sets can be found in Martínez-Jauregui et al. (2023).

For comparisons with the opinions and preferences of the general public, we relied on the sample of Spanish society used by Martínez-Jauregui et al. (2023) and Delibes-Mateos et al. (2023). Briefly, 402 Spanish citizens responded to the survey. They were selected through a consumer panel, and the sample was representative of Spanish society in terms of gender, age, and residence in rural versus urban areas (Martínez-Jauregui et al. 2023; Delibes-Mateos et al. 2023).

### Data analysis

Because accounting for heterogeneity is an important issue in the estimation of choice models, in this study, we applied a latent class model (LCM) with random parameters intra and inter classes (Soliño and Farizo 2014; Soliño et al. 2018; Martínez-Jauregui et al. 2020) to analyze unobserved heterogeneity among respondents (Varela et al. 2014) with the software Latent GOLD® (Haughton et al. 2009). Latent class approaches make use of two sub-models: one for class assignment and one for within-class choice (Hess et al. 2006). The former models estimate the probability of an individual being assigned to a specific class. The within-class model is then used to calculate class-specific choice probabilities for different alternatives conditional on tastes within that class (Hess et al. 2006; Varela et al. 2014).

## Results

We received 98 responses from experts who attended the SECEM conference from 16 out of 17 regions of Spain. Among the respondents, there were 62 men and 36 women and most of them were between 30 and 48 years old (53%). The Spanish society sample (*n* = 402) included 49% were men, 56% were between 29 and 58 years old. Table 2 provides a comparison of this sociodemographic information with the respondents’ data.

**Table 2:** Sociodemographic characterization of respondents

Sociodemographic variables	Categories	Number of responses		Percentage of responses	
		Experts *N* = 98	Society *N* = 402	Experts (%)	Society (%)
Regions	Andalucía	35	80	35.71	19.90
	Aragón	2	12	2.04	2.99
	Baleares	1	2	1.02	0.50
	Cantabria	–	5	–	1.24
	Castilla y León	4	22	4.08	5.47
	Castilla-La Mancha	10	18	10.20	4.48
	Cataluña	10	72	10.20	17.91
	Comunitat Valenciana	5	46	5.10	11.44
	Extremadura	4	10	4.08	2.49
	Galicia	3	25	3.06	6.22
	Comunidad de Madrid	12	57	12.24	14.18
	La Rioja	–	3	–	0.75
	Región de Murcia	3	14	3.06	3.48
	Comunidad Foral de Navarra	3	7	3.06	1.74
	País Vasco	2	20	2.04	4.98
	Principado de Asturias	3	9	3.06	2.24
	No answer	1	–	1.02	–
Gender	Female	36	207	36.73	51.49
	Male	62	195	63.27	48.51
Age	From 1997 to 2003	11	36	11.22	8.96
	From 1987 to 1996	34	60	34.69	14.93
	From 1977 to 1986	18	78	18.37	19.40
	From 1967 to 1976	19	86	19.39	21.39
	From 1957 to 1966	13	67	13.27	16.67
	Before 1956	3	75	3.06	18.66
Place of residence	More than 20 000 inhabitants	70	210	71.43	52.24
	Less than 20 000 inhabitants	28	192	28.57	47.76

### Views on wildlife impacts and wildlife management.

Experts were on average more in agreement than the general public with pollution and/or climate change negatively affecting birds and mammals (*t* = − 7.9; *p* < 0.001), with activities such as hunting, nature sports or other outdoor activities harming birds and mammals (*t* = − 4.36; *p* < 0.001), and with human activities (agriculture, industry, urban developing) changing the habitats of birds and mammals (*t* = − 9.68; *p* < 0.001). Nevertheless, the level of agreement of the general public with these issues was also high; > 70% of the participants agreed with all of these statements (Fig. 1a). We did not detect statistically significant differences between experts and the general public regarding birds and mammals causing damage to other species and their habitats (*t* = − 0.47; *p* > 0.05), birds and mammals causing damage to infrastructure, crops, forests, parks and gardens (*t* = 0.84; *p* > 0.05), birds and mammals transmitting diseases and parasites to livestock and/or pets (*t* = 1.14; *p* > 0.05), and birds and mammals putting human health and safety in danger (*t* = 0.57; *p* > 0.05).

There were differences between experts and the general public regarding their views on wildlife management in three out of four of the statements evaluated. In particular, experts were on average more in agreement than the general public with the need of implementing more management programs to monitor species, increase the benefits to be obtained from them and/or to mitigate the damage their cause (*t* = − 6.69; *p* < 0.001), although the level of agreement among participants of the general public was also high (Fig. 1b). In contrast, the general public was more in agreement than experts with prioritizing management in protected areas (*t* = 7.40; *p* < 0.001), and with the notion that the birds and mammals should not be managed because ecosystems self-regulate (*t* = 7.97; *p* < 0.001). Nevertheless, the level of agreement with the latter was low for both the general public and particularly for experts (Fig. 1b). No statistically significant differences were detected between both groups in their level of agreement with the fact that management programs should be conducted across the whole territory (*t* = − 1.82; *p* > 0.05).

### Experts preferences about wildlife management

The LCM identified five different classes of behavior for the expert sample (Table 3). All five classes had in common a strong preference for wildlife management programs targeting species with scarce populations. For most classes (with the exception of Class 2), the alternative specific constant (ASC) had a significant coefficient value, and in three classes, it had a high positive value, which shows that for most surveyed experts (72.29%) wildlife management is a priority. We found that ASC was the variable with the highest coefficient for 15.47% of the respondents (Class 1*—‘non-urban/non-agricultural’*), indicating that wildlife management is a priority for them. They also favored management programs outside protected areas and in habitats that were neither agricultural nor peri-urban. Class 2 (‘*scarce populations’*) comprised only 8.6% of the respondents, and its only significant attribute was the management of declining species, with the highest coefficient value for this attribute. Although for Class 3 (‘*non-protected areas’*) management programs represented losses (ASC with negative coefficient), the experts in this group (19.11% of respondents were grouped together) showed certain preferences directed toward agricultural habitats and management outside protected areas. For Class 4 (‘*No-PES’*), ASC had the highest coefficient, indicating an improvement in well-being and a preference for management programs that did not include PES (20.42% of the sample). Like the previous class, Class 5 (*‘Forestry-Protect-PES’*) had a high ASC coefficient showing a strong preference for wildlife management programs in forest habitats, within protected areas, and including PES (36.4% of the sample).

**Table 3:** Results of latent class model of experts’ preferences for programs for wildlife management in Spain. Attributes and their levels are presented in Table 1; detailed descriptions are provided in the text. Significance *** *p* < 0.01. **- *p* < 0.05. * *p* < 0.10. Coef.: Coefficient. (s.e.): standard error SDPD: Std. dev. of parameter distributions

	Class 1 ‘*non-urban/non-agricultural*’		Class 2 ‘*scarce population*’		Class 3 ‘*non-protected areas*’		Class 4 ‘*No-PES*’		Class 5 ‘*Forestry-Protect-PES’*		Overall
	Coef. (s.e.)	SDPD	Coef. (s.e.)	SDPD	Coef. (s.e.)	SDPD	Coef. (s.e.)	SDPD	Coef. (s.e.)	SDPD	
OVERAB	− 0.7118 (0.2207)***	0.6312 (0.2779)**	− 7.5825 (3.0587)**	1.771 (1.7079)	− 2.0743 (0.4763)***	1.9882 (0.3996)***	− 2.150 (0.4657)***	− 0.6321 (0.2897)**	− 0.4721 (0.106)***	− 0.1389 (0.1639)	− 0.3259 (0.0886)***
URBAN	− 0.9965 (0.4165)**	− 0.4319 (0.4549)	− 3.6859 (3.8943)	− 7.2183 (4.0346)*	− 0.7849 (0.7136)	0.4166 (0.6042)	0.3227 (0.4755)	− 0.1347 (0.3069)	− 0.5254 (0.149)***	− 0.0243 (0.2506)	− 0.4476 (0.1245)***
AGRIC	− 0.7711 (0.3114)**	0.145 (0.2891)	0.3934 (2.0886)	3.8298 (2.3897)	0.8284 (0.4857)*	− 0.451 (0.411)	− 0.3583 (0.3953)	− 0.5829 (0.3061)*	− 0.100 (0.152)	0.5335 (0.1806)***	− 0.0933 (0.1312)
FOREST	0.3962 (0.3616)	0.4989 (0.4374)	− 1.7883 (2.0828)	3.7099 (1.8493)**	− 0.1886 (0.6083)	− 0.0824 (0.5444)	− 0.5142 (0.4566)	1.0231 (0.4509)**	0.3544 (0.1308)***	− 0.3002 (0.1759)*	0.2384 (0.1337)*
PROTECT	− 0.7921 (0.3464)**	0.4627 (0.3191)	− 1.213 (1.2502)	1.0325 (0.9705)	− 1.0302 (0.477)**	0.7339 (0.3841)*	− 0.5524 (0.3696)	− 0.1357 (0.2293)	0.295 (0.0923)***	0.1205 (0.1465)	0.3443 (0.0822)***
PES	− 0.0791 (0.2061)	− 0.0358 (0.216)	2.3939 (1.9502)	− 0.8552 (1.2862)	0.2039 (0.3629)	0.7886 (0.3168)**	− 0.5908 (0.2971)**	− 0.1426 (0.1946)	0.1461 (0.0736)**	0.2315 (0.1023)**	0.0545 (0.073)
TAX	− 0.0054 (0.0157)	− 0.1013 (0.0237)***	0.0298 (0.1018)	− 0.0855 (0.068)	− 0.0432 (0.0201)**	0.0252 (0.0176)	0.0152 (0.016)	0.0104 (0.0108)	− 0.0145 (0.006)**	− 0.0247 (0.0085)***	− 0.0163 (0.0055)***
ASC	4.7142 (1.2981)***	0.4195 (0.9022)	6.0854 (8.9215)	10.5928 (7.1431)	− 4.1309 (0.9288)***	4.5509 (0.9574)	5.606 (1.801)***	2.0236 (0.8817)	4.9405 (1.0285)***	− 0.9474 (1.0123)	− 2.1423 (0.4504)
R^2^	0.2646		0.6084		0.4608		0.6461		0.6131		0.5144
R^2^(0)	0.3185		0.6086		0.4789		0.6542		0.6203		0.5226
Class size	15.47%		8.60%		19.11%		20.42%		36.40%		100%

### Society preferences about wildlife management

For the general public sample, the LCM also identified five different behavioral classes (Table 4). For all five classes, the alternative specific constant (ASC) had a significant coefficient value, and in four classes, it had a high positive value (with the exception of Class 1), which shows that for most surveyed general public (78.85%) wildlife management is a priority. Class 1 (‘*protected areas’*) grouped 21.15% of respondents and showed preferences for wildlife management programs carried out in peri-urban areas and within protected areas. We found that for 26.99% of the population (Class 2, ‘*non-urban’*), the management of species with scarce populations was a priority, as well as management programs in forest habitats, within protected areas, and outside peri-urban areas. Class 3 (‘*scarce populations,’* 15.18% of the sample) also showed a preference for management of species with low populations, with programs carried out outside agricultural areas, within protected areas and in forest habitats. They also showed a preference for programs involving PES. For 11.07% of the sample (Class 4, ‘*PES*’), ASC was the most important coefficient, followed by their preference for programs that include PES, which take place within protected areas and preferably in non-peri-urban areas. They also favored management programs targeting scarce species. Finally, the last class grouped 36.4% of the respondents (Class 5, ‘*urban*’) who favored wildlife management in peri-urban areas, preferably outside agricultural and forest habitats. They also preferred management programs that target scarce species.

**Table 4:** Results of latent class model of general public’ preferences for programs for wildlife management in Spain. Attributes and their levels are presented in Table 1; detailed descriptions are provided in the text. Significance *** *p* < 0.01. **- *p* < 0.05. * *p* < 0.10. Coef: Coefficient . (s.e.): standard error SDPD: Std. dev. of parameter distributions

	Class1 ‘*protected areas*’		Class2 ‘*non-urban*’		Class3 ‘*scarce population*’		Class4 ‘*PES*’		Class5 ‘*urban*’		Overall
	Coef. (s.e.)	SDPD	Coef. (s.e.)	SDPD	Coef. (s.e.)	SDPD	Coef. (s.e.)	SDPD	Coef. (s.e.)	SDPD	
OVERAB	0.1419 (0.2007)	− 0.0861 (0.1282)	− 0.226 (0.0717)***	− 0.5267 (0.0757)***	− 0.8633 (0.1906)***	0.5846 (0.1542)***	− 0.4443 (0.1635)***	− 0.1855 (0.2662)	− 0.352 (0.1234)***	0.0775 (0.1139)	0.2129 (0.082)***
URBAN	0.6134 (0.2882)**	− 1.0921 (0.2424)***	− 0.4721 (0.1048)***	− 0.3891 (0.1186)***	0.0541 (0.1333)	− 0.2421 (0.1779)	− 0.5477 (0.2526)**	− 0.6848 (0.4236)	0.5338 (0.2063)***	0.5118 (0.1952)	0.1701 (0.083)***
AGRIC	− 0.5369 (0.4191)	1.4919 (0.3267)***	− 0.0492 (0.0735)	− 0.03 (0.0923)	− 0.3033 (0.1466)**	0.1402 (0.1931)	0.3281 (0.2711)	0.466 (0.3856)	− 0.3029 (0.1641)*	0.0637 (0.1436)	− 0.0385 (0.0863)
FOREST	0.2350 (0.2831)	0.132 (0.195)	0.3116 (0.0752)***	0.2106 (0.0974)**	0.268 (0.1315)**	− 0.0748 (0.1569)	0.0262 (0.2431)	− 0.0567 (0.3409)	− 0.3665 (0.188)*	− 0.635 (0.2023)***	− 0.0351 (0.0754)
PROTECT	0.8668 (0.2148)***	− 0.0528 (0.1308)	0.1626 (0.0527)***	− 0.0027 (0.0661)	1.3807 (0.1847)***	0.5973 (0.1471)***	0.5731 (0.154)***	0.3065 (0.2355)	− 0.0912 (0.1228)	− 0.5632 (0.124)***	0.0912 (0.0627)
PES	0.082 (0.1547)	0.0854 (0.1146)	0.0212 (0.0567)	0.2572 (0.0625)***	0.262 (0.1068)**	0.3832 (0.1281)***	0.9038 (0.1439)***	− 0.6479 (0.2044)***	− 0.0179 (0.1384)	− 0.5347 (0.122)***	0.1406 (0.0534)***
TAX	− 0.0832 (0.0124)***	0.0293 (0.009)***	− 0.0128 (0.0035)***	0.0136 (0.0039)***	− 0.0089 (0.0073)	0.0487 (0.0096)***	− 0.1002 (0.0126)***	− 0.165 (0.0215)***	− 0.2356 (0.0196)***	0.1897 (0.0229)***	0.0134 (0.0035)***
ASC	− 2.6238 (0.3705)***	1.8314 (0.3571)***	5.2528 (0.4057)***	0.3496 (0.3003)	4.2327 (0.6632)***	− 1.5345 (0.4397)***	7.131 (0.7557)***	6.4044 (0.9967)***	3.5477 (0.4042)***	0.5652 (0.512)	3.5415 (0.2524)***
R^2^	0.3706		0.721		0.7271		0.3956		0.4166		0.6028
R^2^(0)	0.3756		0.7273		0.7898		0.4234		0.4525		0.6068
Class size	21.15%		26.99%		15.18%		11.07%		25.6%		100%

### Willingness to pay results

**Fig. 2:**
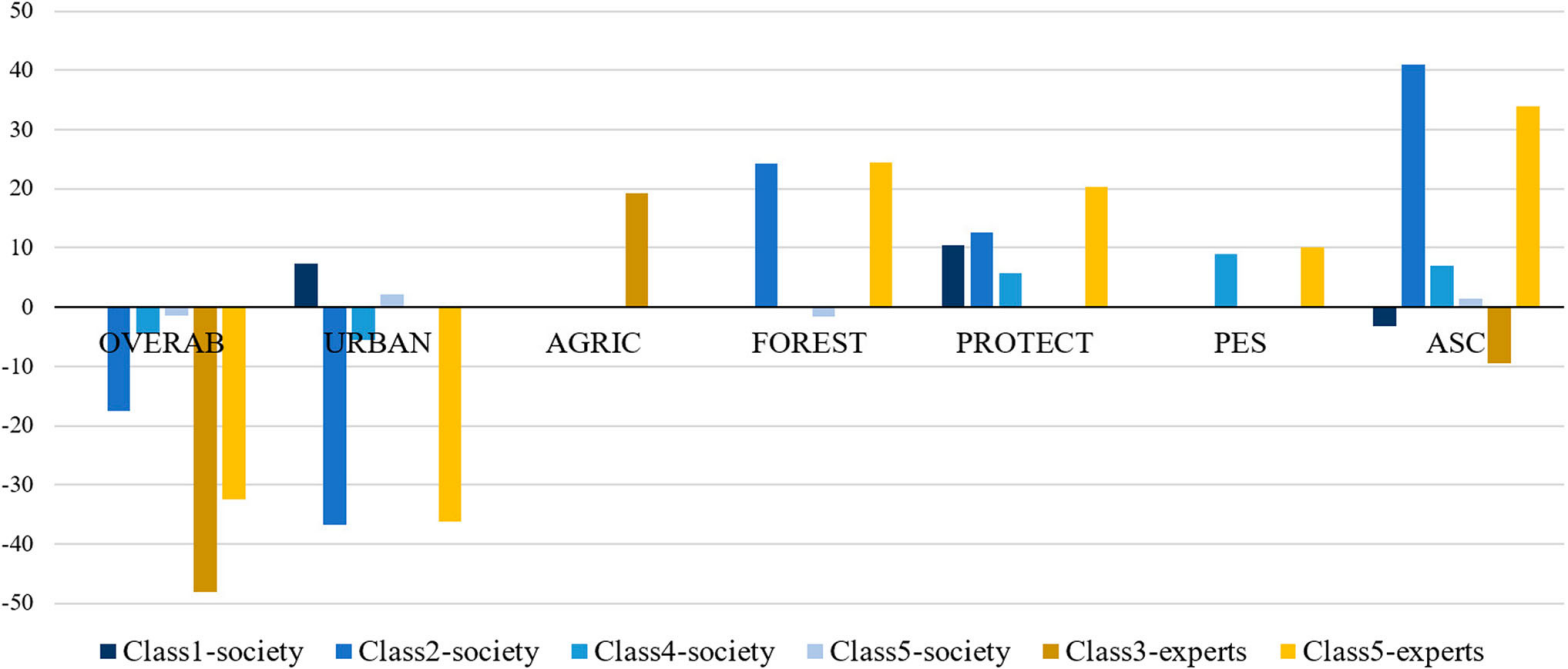
Willingness to pay (WTP, €) for each of the attribute levels considering each latent class that had statistically significant coefficients for cost in the model, in society and expert samples. (Notice that PES means payment for environmental services and ASC is the alternative specific constant of the model, which represents the willingness to pay for carrying out a program without specifying features). *WTP values for the ASC were split into 10 to avoid scaling problems

The values of willingness to pay (WTP; a proxy for change in human well-being) for each of the attributes in each sample (experts and the general public) are shown in Fig. 2. WTP was only calculated for the classes where the estimated coefficient of annual cost of the programs (TAX) was statistically significant at 90% level, that is, classes 3 and 5 of the expert sample and classes 1, 2, 4, and 5 of the general public sample. In general, management programs targeting birds and mammals that increased human well-being showed a clear tendency to address species with scarce populations (with the exception of Class 1-society and Class 3-experts, where a negative constant was obtained in the ASC model). In terms of management areas, a large part of the sample showed a WTP for programs within protected areas. The other attributes did not show such a distinct pattern across classes (Fig. 2).

## Discussion

### Unpacking wildlife concerns

Our results showed a high level of concern, from both experts and the general Spanish public, regarding the increasing negative impacts of human activities on mammal and bird populations. Conversely, they showed a lower level of concern about the impacts of wildlife on people and their livelihoods. Multiple studies have documented the rapid decline in biodiversity in recent decades, reflected in an alarming increase in the global rate of species extinction (De Vos et al. 2015; IPBES 2019). The literature has also shown that wildlife population declines have been caused, in large part, by pressures on wildlife and their habitats from human activities (Rosser and Mainka 2002; IPBES 2022). Although both collectives expressed concern in this regard, it is noteworthy that experts were significantly more aware of the impact of human activities. This greater environmental concern of experts can be conditioned by their social context and the individual’s own characteristics, such as their sense of responsibility, their attitudes toward the environment, their level of commitment, their knowledge of strategies for action in the face of a problem, and especially their level of knowledge and awareness of the problems (Bamberg and Möser 2007; Gifford and Nilsson 2014). People who are more aware of environmental issues, such as experts in our study, tend to be more likely to assess the environmental consequences related to these issues (Dangelico et al. 2022), possibly leading them to show greater environmental concern and, thus, to follow social norms and guidelines for appropriate environmental action (Hirsh 2010).

This result also highlights the need to increase the public's awareness of the current state of biodiversity and the negative consequences of human activities on the natural environment in general. Regarding other environmental issues such as climate change, there has been an improvement in public awareness, knowledge, and concern (Capstick et al. 2015). Although it has been a fluctuating process, it has been shown that putting these topics on political agendas and in the media improves public understanding. There is also evidence of a lack of communication between the scientific community and society, especially regarding wildlife-related issues. In this sense, citizen science is positioned as a tool to engage the general public in biodiversity research and management, promoting their participation in the different phases of knowledge generation while simultaneously creating social capital (Greenwood 2007). The interdisciplinary characteristics of citizen science mean that fields such as education, society, science, and communication work hand in hand. Beyond being an effective method for collecting data and monitoring biodiversity, it contributes to generating useful knowledge for wildlife management and conflict mitigation (Ostermann‐Miyashita et al. 2021).

### Exploring wildlife management scenarios

Our study shows that both Spanish society and experts prioritized the management of scarce species over overabundant species. This is possibly because scarce species, or species with declining populations, tend to be flagship and/or charismatic species that are generally threatened (i.e., included in the IUCN Red List), which results in their conservation programs receiving more financial and social attention (Liordos et al. 2017; Martínez-Jauregui et al. 2023). However, although most conservation efforts are directed at threatened species, widely distributed and abundant species play an important role in ecosystem functioning and the provision of ecosystem services and should therefore be considered within global conservation goals. In addition, overabundant species can affect ecosystems negatively, and impact human well-being (Apollonio et al. 2010). This leads to a shift in the conservation narrative, seeking to move beyond extinction risk as the dominant criterion (Baker et al. 2019).

Previous studies on public preferences for species conservation have often found greater support for animals perceived as attractive, charismatic, and safe, while species associated with fear or negative impacts tend to receive less support (Jarić et al. 2019). However, this pattern is not universal. Several studies have shown substantial willingness to pay (WTP) among citizens for the conservation of large carnivores, despite their potential to generate conflicts, especially when their ecological roles or cultural value are recognized (e.g., Ericsson et al. 2008; Zabel and Holm‐Müller 2008). In our case, the lower support for managing overabundant species may relate to how these species—such as wild boar—are perceived in the Spanish context, where they are often associated with negative impacts on agriculture, biodiversity, and public health (Valente et al. 2020a; Carpio et al. 2024; Dunn‐Capper et al. 2024; De Kruiff et al. 2025). Indeed, the increase in wild boar populations in European countries is currently damaging both the natural environment and people's livelihoods and safety (e.g., damage to agricultural and forestry crops, collisions with vehicles, transmission of diseases to livestock, and habitat disturbance) (Pascual-Rico et al. 2021). This has led to its perception as a problematic species, especially for Iberian society in agricultural and peri-urban contexts (Valente et al. 2024; Alba-patiño et al. under review). Like the wild boar, other ungulates, such as the red deer (*Cervus elaphus*), the roe deer (*Capreolus capreolus*), and the Iberian ibex (*Capra pyrenaica*), have also experienced a considerable increase in their populations in different regions of the Iberian Peninsula in recent decades (Refoyo et al. 2014; Valente et al. 2020b). In these cases, although they are often considered kinder (‘Bambi effect,’ Silk et al. 2018), they have also brought increased attention due to their impacts and associated conflicts (Valente et al. 2020a; Carpio et al. 2024), demonstrating the importance of managing these populations and overabundant wildlife in general. The fact that both public and experts consider this management as less priority highlights the potential conflicts that such management will bring in the future, and the need of further research on this topic (including efficient and acceptable ways of managing those impacts).

Overall, most respondents, both experts and the general public, agreed with the implementation of bird and mammal management programs in Spain, with high ASC values for most classes, indicating an increase in their well-being for 77.6% of the surveyed population. However, when delving deeper into their preferences for specific attributes, clear differences were observed between the two collectives. Spanish society showed a higher WTP for the implementation of wildlife management programs than experts, although the latter showed greater environmental concern. This is contrary to previous studies that have shown that groups with higher environmental concerns may be more willing to take measures to solve environmental problems, including higher WTP (Dangelico et al. 2022). Nevertheless, this behavior may be explained by social identity theory, which holds that an individual’s identification with higher social units (community, nation, and world) strengthens their in-group solidarity and empathy, and consequently, their willingness to protect the environment for the benefit of the group’s well-being (Brieger 2019). In our case, it might be that the manifestation of the social identity of the country in individuals of the Spanish general public influenced their support for the protection of biodiversity and increased their willingness to make economic sacrifices to protect the environment in the interest of group well-being (Brieger 2019). Another possible interpretation of the experts’ behavior is that their active involvement in conservation and their subject-matter expertise make them more skeptical about the experiment itself. They may be more aware of the limitations of stated preference methods. Although we did not observe a higher proportion of status quo choices or protest responses among experts in our sample (only 6% of the sample), future research could investigate more thoroughly how familiarity with policy instruments and their effectiveness influences the stated preferences of expert community.

In addition, for the general public, management in protected areas was prioritized over programs in non-protected areas. As stated in previous studies (Martínez-Jauregui et al. 2021, 2023), this may reflect that people prefer a separation of uses between humans and nature, whereas experts may be inclined to the idea that conservation goes beyond protected areas. It is noteworthy that it is common to find differences between these two collectives regarding their preferences and opinions on wildlife management (Drijfhout et al. 2022; Deparis et al. 2023). However, acknowledging these divergent views should lead to better conservation outcomes, as a way to improve the acceptance of management actions, especially if they are potentially controversial (Martínez-Jauregui et al. 2020; Drijfhout et al. 2022).

It was also relevant to note that experts agreed with the general public in their lower overall preference for management within agricultural habitats (although see also below for within group variation). This is striking given that research has highlighted the loss of farmland biodiversity in Spain as a high conservation priority (Traba and Morales 2019; Lomba et al. 2022). The fact that experts did not consider this management as a priority may reflect a lack of belief that such management could be efficient and that it needs to be applied at large (including outside protected areas) involving a change in farming paradigms. Alternatively, it may indicate skepticism related to the sociopolitical context surrounding the management of agricultural landscapes, which can pose significant challenges to their conservation.

Another key attribute was PES, which revealed particularly diverse responses among experts. While some experts (e.g., Class 4, ‘No-PES’) showed a negative preference for programs including PES, others (e.g., Class 5, ‘Forestry-Protect-PES’) strongly supported them. This heterogeneity suggests that experts' support for PES may depend on the habitat context. Forested areas, often under long-term management frameworks and public–private stewardship, may be perceived as more suitable for implementing PES due to clearer land tenure, governance structures, and measurable conservation outcomes (Engel et al. 2008; Wunder 2015). In contrast, experts may view PES in agricultural or peri-urban contexts with more skepticism, possibly due to implementation challenges, conflicts of interest, or a perception of limited conservation impact (Schomers and Matzdorf 2013). Importantly, both experts and the general public included latent classes that favored PES, indicating that economic incentives may be an acceptable and effective tool across stakeholder groups.

### Differences in preferences within collectives

Our results also identified differences in the preferences for specific management programs within each collective. Interestingly, we observed that even the expert group, despite their in-depth knowledge of the issue, does not speak with a single voice. This highlights the inherent complexity of managing wildlife and their habitats on a global scale, underscoring the challenges of balancing conservation priorities and ecological sustainability. Within the expert collective, classes 3 and 5 favored management programs in agricultural habitats, whereas classes 2 and 4 showed preferences for forest habitats. Management programs outside protected areas were a choice for classes 1 and 3, whereas individuals in Class 5 favored the opposite option. Similarly, the general public also revealed different patterns of preferences. Classes 1 and 5 favored management programs in peri-urban habitats, whereas classes 2 and 3 showed preferences for forest habitats. Likewise, individuals in Class 5 were the only ones who favored programs outside protected areas. Although there may be multiple reasons for these differences, we will explore only a few of them. On the one hand, as mentioned above, even if experts or general public share similar characteristics because they belong to the same collective, individuals’ positions on certain environmental issues can be highly influenced by their personal characteristics such as values toward nature, lived experiences, level of knowledge of the issues, level of commitment, attitudes toward nature, among others (Gifford and Nilsson 2014). On the other hand, in the case of pro-environmental behaviors, it has been found that some people make behavioral choices that reduce environmental damage without the assumed prerequisites. That is, although these people pursue a completely different goal than caring for the environment (e.g., recycling to save money), they provide important co-benefits to the environment (Gifford and Nilsson 2014).

## Conclusions

Although there is no one-size-fits-all solution to wildlife management, understanding how both experts and the general public perceive conservation challenges is essential for developing effective and socially acceptable strategies. Our findings highlight the need to integrate scientific knowledge with public values to design policies that are both evidence-based and widely supported.

The differences observed between experts and the public—particularly in their willingness to pay, management priorities, and the role of protected areas—underscore the importance of fostering dialog and collaboration between actors. Conservation policies should not only be grounded on public perceptions, and our study shows the importance of increasing information toward the general public about the existing problems that may potentially increase their understanding of the need of certain management options and thus their acceptability. Furthermore, conservation policies should be based not only on ecological principles, but also account for social perceptions and economic considerations to enhance their legitimacy, effectiveness, and the long-term success of biodiversity management.

## Supplementary Information

Below is the link to the electronic supplementary material.

Supplementary file1
