# Supplementary material for: Preferences of experts and the general public about wildlife management in Spain
Source: Ambio. 2025 Oct 23;55(4):921–35. doi: 10.1007/s13280-025-02280-z (PMC12960859; doi:10.1007/s13280-025-02280-z)
Supplement: Supplementary file 1 — Supplementary file1 [file 13280_2025_2280_MOESM1_ESM.pdf]

Supplementary Information: This Supplementary Information has not been peer reviewed.

Rationale for the attribute selection

POPULATION SITUATION: The status of the wildlife species reflects two widely recognized priorities in wildlife management: (i) the conservation of rare or declining species, and (ii) the control of overabundant species generating negative impacts (Caughley 1977; Krausman and Cain 2013). These situations are particularly relevant in Europe, where efforts to restore threatened populations and to control expanding species are being developed simultaneously. For example, initiatives such as the EU Nature Restoration Law, the EU Biodiversity Strategy for 2030, or the Spanish Law 42/2007 on Natural Heritage and Biodiversity together with Royal Decree 139/2011 (Jefatura del Estado 2007; Ministerio de Medio Ambiente, y Medio Rural y Marino 2011; European Union 2024), all seek to protect endangered species and restore degraded ecosystems. At the same time, management plans are in place for overabundant species such as wild boar (*Sus scrofa*), whose expansion is associated with ecological, economic and health impacts. An example of this is the European comprehensive plan to manage African Swine Fever (ASF) in wild boar, which includes the Spanish 'National plan for the medium/long-term management of wild boar populations to reduce the risk of the entry and spread of African swine fever virus in Spain', published by the Ministry of Agriculture, Fisheries and Food (2024). Therefore, the inclusion of this attribute in our study allows us to realistically reflect current wildlife management priorities in Europe and analyse how these are valued by society.

HABITATS: The habitat types considered—forest, agricultural, aquatic, and peri-urban—were selected based on their ecological significance and prominence in European conservation frameworks, and therefore yes, they represent most of the habitats present in the area (at least the dominant ones). The Habitats Directive (92/43/EEC) identifies various types of natural forests (such as riparian or Mediterranean sclerophyllous forests) and aquatic habitats (including rivers, lagoons, and wetlands) as priorities for conservation. In addition, the European Biodiversity Strategy 2030, the EU Nature Restoration Law and the Spanish Law 42/2007 on Natural Heritage and Biodiversity explicitly recognise the importance of conserving agricultural and semi-natural habitats (such as grasslands and traditional agricultural systems) in order to conserve associated wildlife species (such as steppe birds as reflected in the 'Strategy for the conservation of threatened birds linked to agro-steppe environments in Spain' Ministerio para la Transición Ecológica y el Reto Demográfico 2022). Finally, the EU Green Infrastructure Strategy (European Commission 2013) recognize the role of peri-urban areas as critical interfaces between human and natural systems. These categories allow for the evaluation of societal preferences across diverse and representative habitat types.

PROTECTED VS NON-PROTECTED: The protected/unprotected area attribute reflects an ongoing debate in both scientific literature and public policy on the most effective spatial strategy for conserving biodiversity. The 'land sparing vs. land sharing' debate (Fischer et al. 2014; Meli et al. 2019) presents two contrasting approaches: one proposes concentrating conservation efforts in protected areas of high ecological value while intensifying other land uses (land sparing); whereas the other promotes integrating

conservation into unprotected areas, favouring connectivity and sustainability at the landscape scale (land sharing). This discussion was also reflected in COP15, where Global Biodiversity Framework proposed protecting at least 30% of terrestrial and marine areas by 2030, opening the debate on what types of areas should be included in this target. In Europe, protected areas are not areas of "wilderness" (as in the US), and the Natura2000 network (one of the most important protection figures in the EU) includes mostly private land, which is therefore economically managed as non protected area. However, protected areas have more limitations about the type of things that can be done in there (in terms of, e.g. land use change or limitation of certain types of management, or chemical use, etc).

PAYMENTS FOR ENVIRONMENTAL SERVICES (PES): PES were included to align with EU and national conservation policy approaches. While the Habitats Directive does not explicitly mention PES, it recognises the importance of economic instruments and voluntary measures to achieve conservation objectives. Furthermore, the relevance of PES in agricultural contexts is explicitly recognised in Spanish conservation policy. In particular, the Strategy for the Conservation of Threatened Birds Linked to Agro-Steppe Environments in Spain (2022) includes PES among the general measures recommended for favourable habitat management. This national strategy expressly mentions 'payments for ecosystem services associated with agrosystems' as actions implemented by various public administrations that have proven beneficial for birds in agricultural environments.

References:

Caughley, G. 1977. Analysis of vertebrate populations. London: Wiley.: 234 pp.

European Commission. 2013. Green Infrastructure (GI) — Enhancing Europe's Natural Capital. European Commission.

European Union. 2024. Regulation (EU) 2024/1991 of the European Parliament and of the Council of 24 June 2024 on nature restoration and amending Regulation (EU) 2022/869. Official Journal of the European Union 1991: 1.

Fischer, J., D. J. Abson, V. Butsic, M. J. Chappell, J. Ekroos, J. Hanspach, T. Kuemmerle, H. G. Smith, et al. 2014. Land Sparing Versus Land Sharing: Moving Forward. Conservation Letters 7: 149–157. doi:10.1111/conl.12084.

Jefatura del Estado. 2007. Ley 42/2007, de 13 de diciembre, del Patrimonio Natural y de la Biodiversidad. Boletín Oficial del Estado.

Krausman, P. R., and J. W. Cain. 2013. Wildlife management and conservation: contemporary principles and practices. JHU Press.

Meli, P., J. M. Rey-Benayas, and P. H. S. Brancalion. 2019. Balancing land sharing and sparing approaches to promote forest and landscape restoration in agricultural landscapes: Land approaches for forest landscape restoration. Perspectives in Ecology and Conservation 17: 201–205. doi:10.1016/j.pecon.2019.09.002.

Ministerio de Agricultura, Pesca y Alimentación. 2024. Plan Nacional De Gestión A Medio/largo Plazo De Las Poblaciones De Jabalíes Silvestres Para Reducir La Entrada Y

Difusión Del Virus De La Peste Porcina Africana - Catálogo De Publicaciones De La Administración General Del Estado (CPAGE). Catálogo de Publicaciones de la Administración General del Estado - CPAGE.

Ministerio de Medio Ambiente, y Medio Rural y Marino. 2011. Real Decreto 139/2011, de 4 de febrero, para el desarrollo del Listado de Especies Silvestres en Régimen de Protección Especial y del Catálogo Español de Especies Amenazadas.

Ministerio para la Transición Ecológica y el Reto Demográfico. 2022. Estrategia de conservación de las aves ligadas a los medios agrarios en España. Ministerio para la Transición Ecológica y el Reto Demográfico.
